# Treatments for Olfactory Dysfunction in COVID-19: A Systematic Review

**DOI:** 10.1055/s-0044-1786046

**Published:** 2024-05-25

**Authors:** Esmaeil Mehraeen, Soudabeh Yarmohammadi, Pegah Mirzapour, Seyed Saeed Tamehri Zadeh, Soheil Dehghani, Leila Molaeipour, Ayoob Molla, Elaheh Karimi, Faeze Abbaspour, SeyedAhmad SeyedAlinaghi

**Affiliations:** 1 Department of Health Information Technology, Khalkhal University of Medical Sciences, Khalkhal, Iran; 2 Trauma Research Center, Kashan University of Medical Sciences, Kashan, Iran; 3 Iranian Research Center for HIV/AIDS, Iranian Institute for Reduction of High-Risk Behaviors, Tehran University of Medical Sciences, Tehran, Iran; 4 Prevention of Metabolic Disorders Research Center, Research Institute for Endocrine Sciences, Shahid Beheshti University of Medical Sciences, Tehran, Iran; 5 School of Medicine, Tehran University of Medical Sciences, Tehran, Iran; 6 Department of Epidemiology, School of Public Health, Iran University of Medical Sciences, Tehran, Iran; 7 School of Medicine, Bushehr University of Medical Sciences, Bushehr, Iran

**Keywords:** COVID-19, SARS-CoV-2, olfactory dysfunction, anosmia

## Abstract

**Introduction** Olfactory dysfunction (OD) has emerged as a notable symptom among coronavirus disease 2019 (COVID-19) patients, with its prevalence varying among different populations. Recognizing the need to provide therapeutic solutions for these individuals, the present study seeks to comprehensively review the current evidence on potential underlying mechanisms and treatment modalities to manage OD in COVID-19 patients.

**Objective** To review the recent evidence on treatments for OD in COVID-19. From the beginning of the study until August 2nd, 2023, we conducted a systematic search on four electronic databases, PubMed, Scopus, Embase, and Web of Science, to find relevant publications.

**Data Synthesis** In the present study, 37 articles were selected for data extraction and included in the final review. The total number of patients was of 3,560 (2,098 female and 1,462 male subjects). The predominant disorders reported were hyposmia, anosmia, and parosmia. In most of the studies, the pre and postintervention assessments were the same, except for one study, in which the pre-intervention assessment of the disorder was through the SST, Sniffin' Sticks Test (SST), and the post-intervention assessment was through the Visual Analog Scale (VAS) and the 22-item Sinonasal Outcome Test (SNOT-22). The findings suggest olfactory training (OT), ivermectin, palmitoylethanolamide, luteolin, and systemic corticosteroids, in combination with topical corticosteroids, are potential therapies for COVID-19 patients with olfactory impairment.

**Conclusion** Although the review suggested several medications for OD treatment, further research must delve into the specific impact of OT, a non-pharmacological modality, regarding the mitigation of OD. By continuing to investigate and refine these therapeutic approaches, we can better support COVID-19 patients and improve their quality of life while navigating the challenges posed by OD.

## Introduction

Changes in the sense of smell are a common phenomenon among patients infected with coronavirus disease 2019 (COVID-19), accounting for up to 40% of all patients. 1 2 3 In most cases, olfactory dysfunction (OD) resolves after several weeks from the infection; nevertheless, it has been shown that nearly 20% of COVID-19 patients develop persistent OD, 4 5 which can have several detrimental impacts on human health, including, but not limited to, depression, social isolation, malnutrition, and death. Therefore, understanding the pathophysiology of OD in COVID-19 patients would be of great help to enhance the quality of life of the affected patients. 6 7 8

The mechanism behind OD is not yet clear; however, several mechanisms, such as obstruction of the olfactory cleft and mucosal thickening, have been proposed as the major ones responsible for OD in the acute phase of the COVID-19 infection. 9 10 11 Metabolic changes in core olfactory and high-order neocortical areas, 12 as well as hypometabolism in the bilateral parahippocampal and fusiform gyri and the left insula 13 of COVID-19 patients have been found, indicating that the virus may cause OD by involving the central nervous system (CNS). It has been hypothesized that molecular mechanisms may play a crucial role in the pathogenesis of OD, since there is a the prevalence of OD in COVID-19 patients varies among different populations, 14 15 and the Omicron variant was found to contribute to a lower OD prevalence compared with the Delta and Alpha variants, which was confirmed in two large cohort studies. 16 17

There is an ongoing debate about the suitable pharmacotherapy for OD treatment. A few clinical trials have demonstrated the short-term beneficial effects of oral or topical corticosteroids; yet, to date, no large study has evaluated their safety and efficacy. 18 Therefore, further studies with larger populations are warranted. Additionally, there is accumulated evidence in support of the fact that olfactory training (OT) can notably improve olfactory function and should be considered in new and existing COVID-19 patients. 19 20

Given this context, in the present review, we discuss what is known regarding the molecular mechanisms involved in the pathogenesis of post-COVID-19 OD and examine the available treatment options for the management of OD as a complication of COVID-19.

## Review of the Literature

### Information Sources and Search Strategies

We systematically searched four electronic databases (PubMed, Scopus, Embase, and Web of Science) to identify relevant articles published until to August 2nd, 2023. Systematic searches were conducted for relevant keywords in the titles and abstracts. Moreover, we examined the reference lists of the extracted articles to identify other relevant publications to review the subject. Supplementary Material 1 provides details of the search strategy.

### Selection Process

**Fig. 1:**
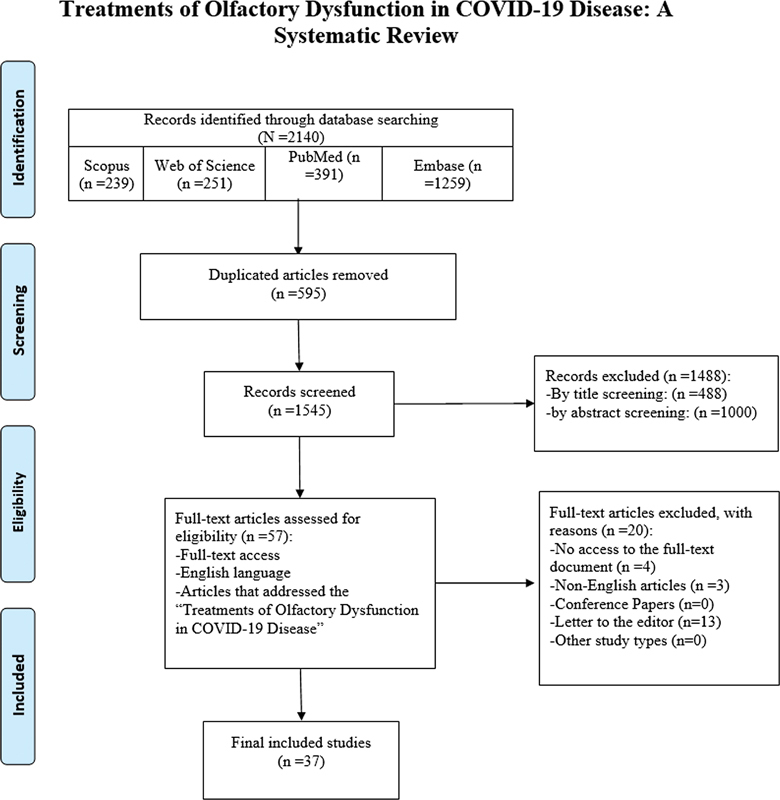
Flow diagram of the current study.

The reference management tool EndNote X9 (Clarivate, London, United Kingdom) was used to import all search results and eliminate any duplicates. The titles and abstracts were independently screened by two authors. Then, two authors read the full text to evaluate them in light of the inclusion and exclusion criteria, with any discrepancies being settled by a third author. A summary of the study selection procedure is presented in a Preferred Reporting Items for Systematic Reviews and Meta-Analyses (PRISMA) flow diagram 21 ( Fig. 1 ).

### Eligibility Criteria

All types of peer-reviewed original literature that addressed the molecular mechanisms behind post-COVID-19 OD and the available management options for the affected patients were included regardless of gender, nationality, race, religion, or publication date. Specific types of publications, namely case reports, editorials, letters, reviews, systematic reviews, and meta-analyses were excluded. Papers published in languages other than English were also excluded, as well as the studies whose full text could not be accessed.

### Data Collection Process

The relevant information, including the first author's name, the year of publication, the country, age and gender of the patients, sample size, study type, disorder type, duration of the disorder, type of preintervention disorder assessment, type and duration of the intervention, improvement rate, type of postintervention disorder assessment, mechanism of treatment, and presence of other diseases were extracted by two authors for each selected study and presented in tables ( Table 1 and 2 ). The present report was formulated according to the PRISMA 2020 statement. 22

**Table 1:** Description of the demographic information of patients

ID	First author (reference)	Country	Type of Study	Sex	Age (years)	Number of patients (N)
1	Abdelalim 27	Egypt	Prospective randomized controlled trial	F = 54; M = 46	29	100
2	Abdelazim 43	Egypt	Prospective randomized double-blinded controlled clinical trial	F = 31; M = 19	Treatment group = 39.25 ± 7.23; control group = 38.37 ± 8.58	50
3	Lechien JR 31	Egypt	Prospective randomized double-blinded controlled clinical trial	F = 30; M = 28	Treatment group = 38.67 ± 7.21; control group = 39.87 ± 6.58	58
4	Saussez S 32	Egypt	Prospective randomized double-blinded controlled clinical trial	F = 38; M = 26	Treatment group = 36.87 ± 5.25; control group = 37.98 ± 6.27	64
5	Abdelmaksoud 44	Egypt	Prospective randomized controlled trial	F = 56; M = 78	N/A	134
6	Marinosci A 34	Egypt	Pilot study	F = 40; M = 20	Treatment group = 28.9 ± 6.31; control group = 30.07 ± 5.74	60
7	Kandemirli 35	Turkey	Prospective randomized controlled trial	F = 52; M = 23	33	75
8	Boscolo-Rizzo P 36	Egypt	Randomized controlled trial	F = 24; M = 72	Treatment group = 30.9 ± 14; control grou= 29.1 ± 9.6	96
9	Gerkin RC 37	Italy	Single-blinded randomized clinical trial	F = 8; M = 4	42.2 ± 14.1	12
10	De Luca 41	Italy	Longitudinal study	F = 43; M = 26	40.6 ± 10.5	69
11	Helman SN 39	Italy	Prospective study	F = 31; M = 14	39.5 ± 12.8	45
12	Kim DH 40	Italy	Prospective randomized controlled trial	F = 70; M = 60	Treatment group = 36.7 ± 11.8; control group = 50.5 ± 12.7	130
13	De Luca P 41	Italy	Multi-center double-blinded randomized placebo-controlled clinical trial	F = 121; M = 64	43.5 + 14.6	185
14	Gracia DI 42	Italy	Double-blinded placebo-controlled multicenter randomized clinical trial	F = 108; M = 94	Control group = 40.9 ± 11.7; once daily PEA–LUT + olfaction training = 42.7 ± 13.5; once daily PEA–LUT = 39.8 ± 11.5; twice daily PEA–LUT = 37.1 ± 13.9	202
15	Pendolino AL 25	Greece	Prospective cohort	F = 89; M = 42	51.45 ± 7.2	131
16	Gracia 42	Spain	Prospective cohort	F = 8; M = 7	40	15
17	Abdelmaksoud AA 44	United States	Triple-blinded, phase 2, placebo-controlled randomized clinical trial	F = 36; M = 15	46 ± 13.1	51
18	Di Stadio A 45	Germany	Prospective case control	F = 54; M = 32	46.9	86
19	Kim DH 40	Egypt	Randomized clinical trial	F = 38; M = 28	39.3	66
20	Abdelalim AA 27	Iran	Prospective double-blinded randomized clinical trial	F = 38; M = 39	32.24	77
21	Rashid RA 46	United States	Single-blinded randomized clinical trial	F = 236; M = 39	41	275
22	Rydzewski B 47	United States	Prospective cohort	F = 39; M = 10	41 ± 16	49
23	Lechien 31	Belgium	Prospective cohort	F = 23; M = 34	40.55 ± 11.66	57)
24	Schepens EJA 26	United Kingdom	Randomized clinical trial	F = 163; M = 26	44	189
25	Schepens EJA 49	United States	Prospective randomized double-blinded placebo-controlled clinical trial	F = 92; M = 25	41	117
26	Zhang AJ 50	Egypt	Randomized clinical trial	F = 39; M = 201	51.9 ± 7.1	240
27	Ono 51	Japan	Retrospective case-control	F = 39; M = 38	37.3	87
28	Pendolino 25	United Kingdom	Cohort study	F = 28; M = 16	40.5	44
29	Pires 52	Brazil	Randomized clinical trial	F = 52; M = 28	36.7 ± 10.3	80
30	Rashid 53	Iraq	Double-blinded randomized placebo-controlled clinical trial	F = 198; M = 78	29	276
31	Saussez 54	Italy	Prospective observational controlled study	F = 40; M = 31	43.5	71
32	Schepens 55	The Netherlands	Double-blinded randomized clinical trial	F = 73; M = 42	48.5	115
33	Schmidt 56	Germany	Randomized clinical trial	F = 14; M = 6	33.9 ± 11.9	20
34	Singh 57	India	Prospective interventional study	F = 32; M = 88	50.88 ± 15.93	120
35	Vaira 58	Italy	Multicenter randomized case-control study	F = 11; M = 9	42.1	20
36	Vandersteen 59	France	Randomized clinical trial	F = 26; M = 17	41	43
37	Yaylacı 60	Turkey	Prospective controlled study	F = 24; M = 19	38 ± 14	51)

**Abbreviations:** F, female; LUT, luteolin; M, male; N/A, not available; PEA, palmitoylethanolamide.

**Table 2:** Description of the findings reported in the eligible studies

ID	Type of disorder	Duration of the disorder	Type of preintervention disorder assessment	Type and duration of intervention	Improvement rate	Type of postintervention disorder assessment	Mechanism of treatment	Other diseases
1	Anosmia and hyposmia	26.41 ± 7.99 days	VAS smell score using familiar substances with a distinctive odor	Mometasone furoate nasal spray in an appropriate dose of 2 puffs (100 μg) once a day in each nostril for 3 weeks	31/50 (62%)	VAS smell score using familiar substances with a distinctive odor	Mometasone furoate nasal spray has no advantages over OT as a topical corticosteroid therapy for the treatment of post-COVID-19 anosmia, which suggests that the pathogenesis is neurological rather than local nasal inflammation	Diabetes (the average time until recovery of the sense of smell was longer in diabetic patients compared with non-diabetic ones)
2	Anosmia	97.37 ± 5.89 days	SST was used to assess the olfactory function by measuring 4 values: threshold (T), discrimination (D), identification (I), and the augmented TDI	An intranasal spray of 1% sodium gluconate, 3 sprays for every nostril 3 times a day for 1 month	20/25 (80%)	SST was used to assess the olfactory function by measuring 4 values: threshold (T), discrimination (D), identification (I), and the augmented TDI	Intranasal sodium gluconate decreased elevated nasal calcium concentration	N/A
3	Anosmia	16.45 ± 1.28 days	SST was used to assess the olfactory function by measuring 4 values: threshold (T), discrimination (D), identification (I), and the augmented TDI	An intranasal spray of 2% nitrilotriacetic acid trisodium salt three times daily for 1 month	N/A	SST was used to assess the olfactory function by measuring 4 values: threshold (T), discrimination (D), identification (I), and the augmented TDI	Intranasal (NTA) can produce a calcium-NTA complex, which lowers the levels of calcium cations in olfactory mucus	N/A
4	Anosmia	94.81 ± 3.89 days	SST was used to assess the olfactory function by measuring 4 values: threshold (T), discrimination (D), identification (I), and the augmented TDI	An intranasal spray of 1% tetrasodium pyrophosphate, 2 sprays for every nostril 3 times a day for 1 month	26/32 (81%)	SST was used to assess the olfactory function by measuring 4 values: threshold (T), discrimination (D), identification (I), and the augmented TDI	Intranasal tetrasodium pyrophosphate remarkably decreased nasal calcium levels	N/A
5	Anosmia and hyposmia	N/A	Anosmia and hyposmia were diagnosed based on the physician's decision and proper examination of the nasal cavity and paranasal sinuse	220 mg zinc sulfate equivocal to 50 mg elemental zinc twice daily	N/A	N/A	Zinc therapy reduced the duration of recovery of olfactory function	N/A
6	Parosmia	More than 3 months	The degree of parosmia was assessed subjectively using the VAS, with scores from 0 to 10	3 platelet-rich plasma injections in the olfactory cleft at 3-week intervals	12/30 (40%) (partial improvement= 9; complete improvement= 3)	The degree of parosmia was assessed subjectively using the VAS, with scores from 0 to 10	It has been shown that platelet-rich plasma promotes axon regeneration and restoration of neurological functions after injury to the peripheral nerves	N/A
7	Parosmia	13.4 months	SST was used to assess the olfactory function by measuring 4 values: threshold (T), discrimination (D), identification (I), and the augmented TDI	MOT with 3 sets of 4 different odors sequentially for 36 weeks. The training was applied for 5 minutes twice a day	N/A	SST was used to assess the olfactory function by measuring 4 values: threshold (T), discrimination (D), identification (I), and the augmented TDI	9 months of MOT helped the patients adjust and improve their scores on odor discrimination tests, which enabled them to identify more odors correctly	N/A
8	Anosmia	19. ± 5.8 days	Anosmia was diagnosed based on the physician's decision. The degree of anosmia was assessed through the VAS, with scoresfrom 0 to 10	Local Ivermectin in the form of nanosuspension mucoadhesive nasal spray (2 puffs per day) for 3 months	47/49 (95.9%)	The degree of anosmia was assessed through the VAS, with scoresfrom 0 to 10	The direct virucidal effect of ivermectin on persistent viral particles or virions on the nasal mucosa and olfactory bulb may be one of the possible mechanisms of treatment	N/A
9	Anosmia and hyposmia	9.7 + 2.5 months	SST was used to assess the olfactory function by measuring 4 values: threshold (T), discrimination (D), identification (I), and the augmented TDI	PEA and luteolin (a daily oral tablet that contained PEA 700 mg and luteolin 70 mg) for 1 month	N/A	SST was used to assess the olfactory function by measuring 4 values: threshold (T), discrimination (D), identification (I), and the augmented TDI	PEA may reduce olfactory bulb inflammation by modifying microglia's polarization in the M2 (name of one kind of macrophage) protective phenotype, promoting neural regeneration, and even recovery of smell. Luteolin can block the polarization of bad microglia and regulate transcription factors like STAT3, NF-κB, and AP-1, preventing brain cell degeneration and reducing inflammation	N/A
10	Parosmia	Previously-trained group (PEA-LUT plus OT) = 8.8 ± 2.6 months; training-naïve 1 (PEA-LUT plus OT) = 8.5 ± 1 months; training-Naïve 2 (PEA-LUT alone) = 8.4 ± 1.7 months	SST was used to assess the olfactory function by measuring 4 values: threshold (T), discrimination (D), identification (I), and the augmented TDI	Ultra-micronized PEA and luteolin (a daily oral tablet that contained PEA 700 mg and louteolin 70 mg) for 3 months	N/A	SST was used to assess the olfactory function by measuring 4 values: threshold (T), discrimination (D), identification (I), and the augmented TDI	PEA exerts an anti-inflammatory effect by modulating histamine release, reducing mast cell degranulation, and activating M2 (name of one kind of macrophage) microglia, which leads to the recovery of olfactory pathways. Luteolin shows anti-inflammatory properties by reducing intracellular reactive oxygen species	N/A
11	Anosmia and hyposmia	More than 180 days	SST was used to assess the olfactory function by measuring 4 values: threshold (T), discrimination (D), identification (I), and the augmented TDI	Ultra-micronized PEA-LUT plus OT for 3 months	32/45 (71.2%)	SST was used to assess the olfactory function by measuring 4 values: threshold (T), discrimination (D), identification (I), and the augmented TDI	Ultra-micronized PEA-LUT modulates mastocyte activation and neuroinflammation process	N/A
12	Anosmia, hyposmia, and parosmia	8.8 ± 3.7 months	SST	Ultra-micronized PEA and luteolin (a daily oral tablet that contained PEA 700 mg and luteolin 70 mg) for 3 months	Improvement rates were not reported for anosmia and hyposmia; however, 58/94 (61.7%) cases with parosmia recovered	SST	Ultra-micronized PEA and luteolin is effective to treat brain neuro-inflammation, which is the main responsible for quantitative smell disorders, but it has little to no effect on peripheral damage (neuro-epithelial, olfactory nerve), which is the cause of qualitative disorders	N/A
13	Anosmia and hyposmia	8.4 ± 2.9 months	SST was used to assess the olfactory function by measuring 4 values: threshold (T), discrimination (D), identification (I), and the augmented TDI	Ultra-micronized PEA and luteolin (a daily oral tablet that contained PEA 700 mg and luteolin 70 mg) for 3 months	120/130 (92%)	SST was used to assess the olfactory function by measuring 4 values: threshold (T), discrimination (D), identification (I), and the augmented TDI	Ultra-micronized PEA and luteolin was supposed to enhance regeneration during OT by lowering the level of COVID-induced neuroinflammation. The PEA component regulates microglial polarization to a protective M2 phenotype, promoting neuronal repair and smell recovery. Luteolin inhibits brain cell degeneration by preventing the polarization of pro-inflammatory microglia	N/A
14	Anosmia and hyposmia	8.8 ± 3.4 months	SST was used to assess the olfactory function by measuring 4 values: threshold (T), discrimination (D), identification (I), and the augmented TDI	Ultra-micronized PEA and luteolin (a daily oral tablet that contained PEA 700 mg and Luteolin 70 mg) for 3 months	89.2%	SST was used to assess the olfactory function by measuring 4 values: threshold (T), discrimination (D), identification (I), and the augmented TDI	Ultra-micronized PEA and luteolin could support the neuroplastic alterations of OT by providing a more favorable regeneration environment. PEA-LUT's anti-neuroinflammatory properties minimize inflammation in the olfactory bulbs and enable normal immature neuron formation	N/A
15	N/A	3 weeks	SST	A: oral steroid course (14 days) + OT (16 weeks); B: OT (16 weeks)	A: 43/78; B: 26/53	SST	Steroids improve underlying upper airway inflammatory conditions which are not related to the causative infection of olfactory loss	Contraindications for oral steroids (including uncontrolled diabetes, osteoporosis, and high blood pressure)
16	N/A	12 to 24 months	SST + EEG	N/A	N/A	SST + EEG	N/A	N/A
17	N/A	3 to 12 months	UPSIT	A: theophylline -400 mg twice a day + nasal irrigations (6 weeks); B: placebo + nasal irrigations (6 weeks)	A: 11/22; B: 6/23	UPSIT	A phosphodiesterase inhibitor promotes neural olfactory signaling and sensory axonal regeneration by preventing the breakdown of important secondary messengers cyclic adenosine monophosphate and cyclic guanosine monophosphate	N/A
18	N/A	8.2 months	SST	A: topical administration of mometasone -100 µg twice a day + OT (3 months); B: OT (3 months)	N/A	SST	N/A	N/A
19	Anosmia	Beyond 3 months after negative SARS-COV test	SST + obtaining nasal secretion to measure the concentration of calcium cations	A: 0.9% sodium chloride nasal spray (3 times a day for 1 month); B: 2% DTPA nasal spray (3 times a day for 1 month)	N/A	SST + obtaining nasal secretion to measure the concentration of calcium cations	Calcium cations are necessary for smell transmission through an inhibitory feedback inhibition approach, and DTPA can chelate calcium cations in an alkaline pH medium, suggesting its use in patients with post-COVID-19 infection	N/A
20	Anosmia	2 weeks	VAS and the UPSIT	A: mometasone furoate 0.05% nasal spray (2 puffs twice a day for 4 weeks) + OT; B: topical saline spray (2 puffs twice a day for 4 weeks) + OT	N/A	VAS and the UPSIT	N/A	N/A
21	Current olfactory loss	2 weeks	UPSIT + CGI-I self-report improvement scale); + ODOR olfaction-related quality-of-life questionnaire)	OT (sniff twice a day -for 3 months; 4 different odors)	Intervention: 56/240; control: 5/35	UPSIT + CGI-I (self-report improvement scale) + ODOR (olfaction-related quality-of-life questionnaire).	N/A	N/A
22	Hyposemia and anosmia	N/A	UPSIT + CGI	Watch and wait for spontaneous recovery (for 6 months)	N/A	UPSIT + CGI	N/A	N/A
23	Persistent olfactory dysfunction	3 months	SST	OT (15.4 weeks)	Adhering to OT: 12/2; not adhering to OT: 14/25	SST	N/A	N/A
24	N/A	At least 4 weeks	Brief Smell Identification Test + Taste Strips + self-rating of smell and taste function questionnaire	OT (12 weeks)	N/A	Brief Smell Identification Test + Taste Strips + self-rating of smell and taste function questionnare	N/A	N/A
25	Persistent olfactory dysfunction	200 days	Brief Smell Identification Test	Omega-3 fatty acid supplementation (2000mg daily- 6 weeks)	N/A	Brief Smell Identification Test + QOD-NS and SNOT-22	High levels of o mega-3 fatty acids are associated with neuro-regeneration and reduced cellular inflammation	N/A
26	Anosmia and hyposmia	10.7 days	Butanol threshold + discrimination tests	A: combination therapy – antihistamine and corticosteroid nasal spray (3 weeks); B: antihistamine nasal spray (3 weeks); C: corticosteroid nasal spray (3 weeks; D: saline nasal spray (3 weeks)	N/A	Butanol threshold + discrimination tests	Reducing inflammation and edema due to reduced eosinophilic inflammation in the olfactory region and improved symptoms of allergic rhinitis + antihistamines minimize the histamine-related cytokine storm	N/A
27	N/A	N/A	NRS scores for OD	A: Japanese traditional Kampo medicine; B: Western medication	N/A	NRS scores for OD	Anti-inflammatory and immunomodulatory effects, enhanced circulation, and nerve protection	N/A
28	N/A	N/A	SST	A: combination therapy –prednisolone 40 mg a day + nasal drop betamethasone (2 weeks) + OT; B: OT; C: no treatment	N/A	VAS + SNOT-22	N/A	N/A
29	N/A	At least 4 weeks	VAS + UPSIT	A: advanced OT (4 weeks); B: classic OT (4 weeks)	N/A	VAS + UPSIT	Adding more scents cannot improve the OD	N/A
30	Anosmia	4.5 days	N/A	Intervention: drop nasal betamethasone 3 times a day (maximim = 1 month); Placebo: drop sodium chloride 9% (maximum = 1 month)	83% of participants had recovered from anosmia (82% in the intervention group and 84% in the placebo group)	Self-report	Slower recovery in the intervention group shows that corticosteroids could impede the regeneration of olfactory epithelium	N/A
31	Hyposmia, anosmia, and parosmia	60 days	SST	OT with oral and nasal corticosteroids, 60 days	N/A	SST	N/A	N/A
32	N/A	12 weeks	SST	Oral prednisolone treatment of 40 mg once a day for 10 days was received. The olfactory function was evaluated 12 weeks after the start of the treatment	N/A	TDI, TST, ODQ, and self–reported with VAS	N/A	N/A
33	Hyposmia, anosmia, and parosmia	5 months	SST	Topical nasal corticoid, OT contains 4 international standard perfumes (rose, lime, eucalyptus, and clove) which the patients smelled twice a day for at least 5 minutes	N/A	SST	N/A	N/A
34	Anosmia and dysgeusia	5 days	N/A	Fluticasone nasal spray and triamcinolone oral paste	N/A	N/A	N/A	N/A
35	Anosmia and hyposmia	40 days	CCCRC	Systemic prednisone and nasal irrigation with betamethasone, ambroxol, and rinazine were administered for 15 days. Olfactory performance was evaluated on the 20th and 40th days.	N/A	CCCRC	N/A	N/A
36	Dysosmia	3.5 months	SST and the short version of the ODQ	Patients should perform OT with olfactory kits impregnated with dill, thyme, cinnamon, cloves, coriander leaves, vinegar, cumin, lavender, coffee, vanilla, or mint twice a day for 6 months	N/A	SST and the short version of the ODQ	N/A	N/A
37	Parosmia	12 weeks	SST	The patients were offered fragrances (lemon, rose, clove, and eucalyptus). Patients must be exposed to each odor twice a day for 12 weeks.	N/A	SST	N/A	N/A

**Abbreviations:** AP-1, activating protein-1; CCCRC, Connecticut Chemosensory Clinical Research Center test; CGI-I, Clinical Global Impressions–Improvement; COVID-19, coronavirus disease 2019; DTPA, diethylenetriamine pentaacetate; EEG, electroencephalography; LUT, luteolin; MOT, modified olfactory training; N/A, not available; NF-κB, nuclear transcription factor-κB; NRS, Numerical Rating Scale; NTA, nitrilotriacetic acid trisodium salt; OD, olfactory dysfunction; ODOR, Olfactory Dysfunction Outcomes Rating; ODQ, Olfactory Disorders Questionnaire; OT, olfactory training; PEA, palmitoylethanolamide; QOD-NS, Questionnaire of Olfactory Disorders - Negative Statements; SARS-CoV, severe acute respiratory syndrome coronavirus; SNOT-22, 22-item Sinonasal Outcome Test; SST, Sniffin' Sticks Test; STAT3, signal transducer and activator of transcription 3; TDI, Threshold, Discrimination, Identification scores; TST, Taste Strip Test; UPSIT, University of Pennsylvania Smell Identification Test; VAS, Visual analogue scale.

### Risk of Bias Assessment

The revised Cochrane Risk of Bias Tool for Randomized Trials, version 2.0 (RoB 2), was used to assess the risk of bias of randomized controlled trials (RCTs). 23 In addition, to assess observational studies (cohort and case-control studies) for potential biases, the Newcastle-Ottawa Scale (NOS) was employed. 24 The risk-of-bias assessments were conducted independently by two authors. To achieve consensus, a third author was recruited to resolve any disagreements ( Table 3 ).

**Table 3:** Risk of bias assessment according to the Newcastle-Ottawa Scale (NOS)

ID	First author	Selection (out of 4)	Comparability (out of 2)	Exposure/Outcome (out of 3)	Total (Out of 9)
1	Abdelalim 27	4	1	3	8
2	Abdelazim 43	4	1	3	8
3	Abdelalim AA 27	3	1	3	7
4	Hummel T 28	3	1	3	7
5	Abdelmaksoud 44	4	1	3	8
6	von Bartheld CS 30	4	2	3	9
7	Kandemirli 35	4	1	3	8
8	Saussez S 32	4	1	3	8
9	Parma V 33	3	1	3	7
10	De Luca 41	4	1	3	8
11	Bilinska K 35	3	1	3	7
12	Boscolo-Rizzo P 36	4	1	3	8
13	Gerkin RC 37	4	1	3	8
14	Gerkin RC 38	4	1	3	8
15	Helman SN 39	3	2	3	8
16	Gracia 42	4	2	3	9
17	De Luca P 41	4	2	3	9
18	Gracia DI 42	3	2	3	8
19	Pendolino AL 25	4	2	3	9
20	Abdelazim MH 43	4	2	3	9
21	Abdelmaksoud AA 44	3	2	2	7
22	Di Stadio A 45	4	2	3	9
23	Lechien 31	4	2	3	9
24	Abdelalim AA 27	3	2	3	8
25	Rashid RA 46	3	2	2	7
26	Rydzewski B 47	4	2	3	9
27	Ono 51	4	1	3	8
28	Pendolino 25	4	2	3	9
29	Pires 52	4	1	3	8
30	Rashid 53	4	2	3	9
31	Saussez 54	3	1	3	7
32	Schepens 55	3	2	3	8
33	Schmidt 56	4	2	3	9
34	Singh 57	3	2	2	7
35	Vaira 58	4	1	2	7
36	Vandersteen 59	4	1	3	8
37	Yaylac 60	3	2	3	8

In the initial database search, 2,140 articles were retrieved, 595 of which were duplicates. The remaining 1,545 articles were screened considering the inclusion and exclusion, and ultimately 37 articles were selected for the final analysis and data extraction. Fig. 1 illustrates the details of the article selection process.

In the present review, most of the studies were from Egypt ( *n* = 9), Italy ( *n* = 8) and the United States ( *n* = 4). Turkey, Germany, and the United Kingdom were represented by two studies each, and Greece, Spain, Iran, Belgium, Japan, Brazil, Iraq, The Netherlands, India, and France each contributed with one study. The total number of patients examined in these studies was of 3,560, (2,098 female and 1,462 male subjects) ( Table 1 and 2 ).

Regarding the types of disorders, most were issues related to hyposmia, anosmia, and parosmia. In most of the studies, the pre and post-intervention disorder assessments were the same, and were performed using the Visual Analogue Scale (VAS), the Sniffin' Sticks Test (SST), electroencephalography (EEG), the University of Pennsylvania Smell Identification Test (UPSIT), the Clinical Global Impressions–Improvement (CGI-I) scale, the Numerical Rating Scale (NRS), the Connecticut Chemosensory Clinical Research Center (CCCRC) test, among others, except for one study 25 in which the preintervention disorder assessment included the SST, and the postintervention disorder assessment was performed through the VAS and the 22-item Sinonasal Outcome Test (SNOT-22), and another study 26 in which the preintervention assessment was performed using the SST and, after the intervention, the Threshold, Discrimination, Identification (TDI) scores, Taste Strip Test (TST), the Olfactory Disorders Questionnaire (ODQ), and the self-reported VAS score. In the investigations, it was found that the average time for recovery of the sense of smell was longer in diabetic patients compared with non-diabetic ones. 27 More details of this review are presented in Tables 1 and 2 .

Among the pharmacological therapies, the combination of palmitoylethanolamide (PEA) and luteolin was the most common intervention evaluated in the included studies. Topical and systemic corticosteroids, local ivermectin, herbal remedies, platelet-rich plasma injection, zinc sulfate, theophylline, and omega-3 were the other interventions assessed in the studies. Moreover, OT, which is described as exposure twice a day to a set of four odors, including rose, eucalyptus, lemon, and cloves, from media such as brown jars or markers, 28 was the most frequent non-pharmacological intervention applied in the included studies.

## Discussion

The precise prevalence of OD caused by COVID-19 is difficult to establish; it depends on the severity of the disease, the geographic region, and the technique of measuring. Two recent systematic reviews 29 30 have reported prevalence of loss of smell ranging from 43% to 62%. Additionally, data from sizable European cohorts 31 32 indicate prevalence rates between 50% to 85%. Based on the current evidence, Europe and North America are the regions with the highest prevalence rates.

There is still no clear understanding of how the severe acute respiratory syndrome coronavirus 2 (SARS-CoV-2) virus causes olfactory impairment. 4 Numerous viruses produce conductive olfactory dysfunction, along with nasal congestion, inflammation, and rhinorrhea, which prevents individuals from detecting odors during the acute stage of the infection. These symptoms are less frequent in COVID-19 and, when they occur, they do not accurately reflect the level of olfactory impairment. 33 The symptoms may also result from potential injury to or death of olfactory neurons or cells in the olfactory bulb; however, since most people who experience loss of smell due to COVID-19 recover quickly, this is less likely, because olfactory neurons lack angiotensin-converting enzyme 2 (ACE2) receptors, which enable viral entrance into cells. The ACE2 receptors and the supporting components for olfactory neurons 34 can be detected in the olfactory epithelium. Notably, the olfactory epithelium's sustentacular cells, which are essential for olfactory neuron functionality, can become infected, 35 suggesting that their inflammation and infection could adversely affect olfaction.

Many people with COVID-19-related olfactory impairment only experience transient symptoms, and they quickly regain their normal sense of smell. 9 10 Some studies have indicated full recovery within two to four weeks. 31 36 However, for a subset of patients, olfactory issues persist even after other COVID-19 symptoms have resolved. According to data from the Global Consortium of Chemosensory Research, 37 up to 50.7% of people may continue to experience olfactory impairment 40 days after the initiation of COVID-19. Given the prevalence of infections (> 295 million infections worldwide as of December 2021) and the 5% to 7% of subjects who were found to be functionally anosmic 12 months after exposure, ∼ 15 million people could develop persistent anosmia, carrying a sizable burden of OD and a long-term disruption in quality of life. 36 38

Highlighting the frequent remission of olfactory impairment within a month of the COVID-19 infection, we find it crucial to distinguish this in the evaluation of preventive and therapeutic strategies. Unlike previous systematic reviews composed mostly of RCTs, 29 39 40 the current review is more inclusive, encompassing three prospective or longitudinal cohort studies that track participants over time. 25 41 42

In the present analysis, treatments such as topical and systemic corticosteroids, OT, local ivermectin, PEA, and luteolin seemed promising. In contrast, herbal remedies, platelet-rich plasma injection, zinc sulfate, theophylline, and omega-3 appeared to be ineffective. 43 44 45 Using PEA and luteolin, which are effective treatment options in two studies, 36 37 in combination with saline irrigation may help this situation progress faster, because the delivery of medication to the olfactory cleft may be increased compared with that of standard nasal spray administration, and this may be further enhanced by using particular head positions. 25 40 Luteolin and PEA may act to lessen nasal cavity inflammation and hasten the process of epithelium regeneration. The present review suggests that the exclusive use of systemic corticosteroids might mirror conditions such as diabetes mellitus and prolonging OD recovery. The underlying processes for these conditions seem interconnected, attributed to compromised immune cell function and diminished capacity to repair the olfactory epithelium. 27 46

The findings of the present study suggest that the use of topical corticosteroids can expedite recovery from COVID-19-induced OD within 2 to 4 weeks post-treatment. These findings are consistent with the theory that olfactory impairment in COVID-19 is primarily a result of an inflammatory process in the olfactory epithelium, in which intranasal corticosteroids might provide beneficial anti-inflammatory effects. 46 Intranasal corticosteroids reduce local inflammation and may also improve olfaction by changing the activity of olfactory receptor neurons due to their effects on the sodium/potassium adenosine-triphosphatase Na ^+^ /K ^+^ -ATPase enzyme. 47 It is interesting to note that two of the trials in the current study demonstrated that combining systemic steroids with OT was more beneficial than using systemic steroids alone. 32 It should be mentioned that, despite the potential advantages of these pharmacotherapies, a recent position paper on OD 48 emphasizes the lack of high-level evidence to support any pharmacologic treatment in the management of OD.

Despite the comprehensive insight that the present review offers on OD treatment in COVID-19, some limitations should be considered in the interpretation of the results. First, the included studies used various techniques and medication treatments for olfactory rehabilitation, potentially leading to heterogeneous results; however, they are probably minimal, as the bulk of the research methods were comparable across the intervention and control groups, and the pre- and post-intervention treatments were identical, except for one study. 26 There were also discrepancies in intervention timing, quantity, and dosage across trials. Large-sample RCTs and prospective cohort studies are essential to validate our findings. The reliability of our evidence is generally low, mainly due to the limited sample sizes in single studies and potential performance bias from a lack of participant blinding.

Our data are supported by anecdotal links between COVID-19-induced OD, inflammation in the olfactory cleft, and magnetic resonance imaging (MRI) evidence of viral infiltration into the olfactory bulb. 49 50 We believe that by reducing mucosal inflammation and olfactory cleft blockages, nasal steroids can aid in olfactory rehabilitation. However, this remains a topic for debate. While combining saline irrigation with treatments like PEA and luteolin might accelerate recovery, OT remains a viable strategy, especially when paired with other therapies.

## Conclusion

Coronavirus disease 2019 has been found to cause anosmia and hyposmia more frequently than other viral infections of the upper respiratory tract. This not only has emotional ramifications for patients but also reduces their ability to detect environmental dangers such as fires and gas leaks. Consequently, doctors must have effective treatments available for patients presenting with COVID-19-related olfactory impairments. For an enhanced quality of life, it is vital to devise strategies that can expedite the disease's resolution. The current research indicates that OT, ivermectin, PEA, luteolin, and systemic corticosteroids, when paired with topical corticosteroids, emerge as potential treatments for those suffering from olfactory deficits due to COVID-19. Future studies should particularly explore the efficacy of OT, a non-pharmacological intervention, in addressing this concern.
